# Conditional Cell Reprogramming and Air–Liquid Interface Modeling Life Cycle of Oncogenic Viruses (HPV and EBV) in Epithelial Cells and Virus-Associated Human Carcinomas

**DOI:** 10.3390/v15061388

**Published:** 2023-06-17

**Authors:** Abdul Qawee Rani, Dilber Nurmemet, Joseph Liffick, Anam Khan, Darrion Mitchell, Jenny Li, Bo Zhao, Xuefeng Liu

**Affiliations:** 1 Comprehensive Cancer Center, Ohio State University, Columbus, OH 43210, USA; 2 Department of Radiation Oncology, Wexner Medical Center, Ohio State University, Columbus, OH 43210, USA; 3 Division of Infectious Diseases, Department of Medicine, Brigham and Women’s Hospital, Harvard Medical School, Boston, MA 02115, USA; 4 Departments of Pathology, Urology and Radiation Oncology, Wexner Medical Center, Ohio State University, Columbus, OH 43210, USA

**Keywords:** conditional cell reprogramming, EBV, HPV, oncogenic viruses, air–liquid interface

## Abstract

Several oncogenic viruses are associated with approximately 20% of human cancers. Experimental models are crucial for studying the pathogenicity and biological aspects of oncogenic viruses and their potential mechanisms in tumorigenesis. Current cell models have considerable limitations such as: their low yield, genetic and epigenetic modification, and reduction in tumor heterogeneity during long propagation. Cancer cell lines are limited and not appropriate for studying the viral life cycle, for example, natural viral life cycles of HPV and EBV, and their persistence and latency in epithelial cells are poorly understood, since these processes are highly related to epithelial differentiation. Therefore, there is an urgent need of reliable human physiological cell models to study viral life cycle and cancer initiation. Conditional cell reprogramming (CCR) is a rapid and robust cell culture system, where the cells can be established from minimally invasive or noninvasive specimens and their lineage functions preserved during the long-term culture. These CR cells retain their ability to differentiate at air–liquid interface (ALI). Here, we recapitulated the applications of CR and ALI approaches in modeling host–virus interactions and viral-mediated tumorigenesis.

## 1. Introduction

### 1.1. Viruses and Cancer

There are seven human viruses that are recognized as being associated with several types of human cancer and are estimated to cause up to 20% of all cancer cases worldwide [1]. Human oncogenic viruses include human papillomavirus (HPV), Epstein–Barr virus (EBV), human T-cell lymphotropic virus-1 (HTLV-1), human herpesvirus-8 (HHV-8), Merkel cell polyomavirus (MCPyV), and hepatitis B and C viruses (HBV and HCV).

EBV and HPV both infect and replicate in upper aerodigestive tract epithelia, in the suprabasal compartment of stratified epithelium. Their life cycles, where lytic reactivation of EBV and the productive phase of the HPV, are both induced upon differentiation of the stratified epithelium [2,3].

Many studies were conducted to understand the molecular interplay in the progression of human cervical cancer from the oncogene [4,5] to tumor suppressor genes [6,7] and its association with HPV [8,9]. HPV type 16 and 18 are the cause of approximately 75% of cervical cancer cases, while HPV type 31 and 45 are the causes of 10% of cervical cancer worldwide [10].

Several viral oncogenic direct mechanisms were suggested, such as generation of genomic instability, alterations in DNA, resistance to apoptosis, cell polarity repair and an increase in the rate of cell proliferation. These mechanisms are often associated with immune evasion of the antiviral immune response. The viral cellular transformation effect takes place if the virus is inside the host cell and can be due to expression of viral oncogenes simultaneously with deregulation of cellular tumor suppressor genes and/or oncogenes.

Viral agents may contribute indirectly to cancer initiation when it is outside of the cell, through chronic antigenic stimulation [11] and chronic inflammation, which produce mutagenic molecules that cause damage to the surrounding tissues or immunosuppression with loss of the cancer immunosurveillance mechanisms [12,13,14,15].

However, the main mechanism of cell transformation by viruses could be due to persistent inflammation of the tumor microenvironment, since the inflammatory molecules contribute significantly to tumor initiation and progression [16,17]. Viruses are also capable of modifying the properties of an established tumors [18].

HPV and EBV can initiate cancer via different signaling transmission pathways such as PI3K/Akt and ERK/MAPK that involve several active molecules such as Akt (pAkt) and ERK (pERK) [19,20].

### 1.2. Cancer Models

Cancer is not a single homogenous and distinct tumor system, but a heterogeneous and highly variable system. The progress of oncology research relies on models that capture the morphological, molecular, and functional characteristics of patient tumors. Although many models were developed and extended to all new phases of drug discovery including target screening, tolerability, toxicity, biomarker discovery, and personalized medicine, choosing an appropriate model to best reflect a given tumor system is still complicated [21].

Employing cell lines for preclinical research has the potential for less limitations. Primary tumor-derived cells grown on plastic may have a different rate of growth and during the long propagation, the cell clone that grows fastest will dominate the culture [22]. The architecture of the tumor tissue might be significantly modified due to alteration of the growth rate, genetic modification along the propagation, and diminution of tumor heterogeneity. This may affect the complex interaction between the tumor and the surrounding microenvironment and reduce the relevance of these models to preclinical applications.

In addition to cancer cell lines and three-dimensional (3-D) model organoids, there are several experimental models for human cancer research including computational cancer models and a few organisms such as patient-derived xenografts (PDXs), zebrafish, genetically engineered pigs and mice, and *Drosophila melanogaster.*

Patient-derived cancer models (PDCMs) represent the next generation of cancer models, and provide an important tool toward more understanding of viral biology and cancer and to drive clinical decision-making. To obtain PDCMs, several approaches were developed, including induced pluripotent stem cells (iPSCs), 3-D organoids, patient-derived xenografts (PDXs), and conditionally reprogrammed cells (CRCs). These models serve as a basis to study cancer pathology, genetic, or biochemical pathways [23]. Cancer models provide cumulative information to increase our knowledge of the subtleties of cancer development in greater details.

## 2. Current Problem with Models and Cell Lines of Virus-Mediated Tumors

In addition to the perturbation gained from the original tumors, primary cells may accumulate abnormalities in the genetic and epigenetic characteristics during the long-term propagation. Furthermore, cellular senescence limits their population doubling and life span [24]. For example, HeLa cells, the first human immortalized cell line and commonly used in scientific research [25] derived from cervical cancer tissue were used for in vitro research for more than seventy years. Nevertheless, drawing conclusions to clinical oncology is still challenging.

PDXs can retain their genetic diversity and intratumoral clonal architecture after repeated passaging [26]; therefore, it serves for diagnostic drug screens and precision medicine [27]. However, their slow generation and high-cost limit their utility in research. CCR coupled with air-liquid interface (ALI) might overcome the limitations of the conventional PDCMs techniques, since the technology is simple, rapid and efficient to present ex vivo models from a wide variety of cell sources and maintains their natural structure and arrangement, physiologic and dynamic processes such as mucus secretion of airway tissues, polarized cellular and junctional properties, subcellular localization, and physiological expression of characteristic proteins [28,29,30,31,32].

To the best of our knowledge, there are no available cell lines from HPV-mediated cancers such as: vulvar, anal, or penile lesions and cancers in the field due to a lack of models of natural HPV infection and the complexity of the HPV life cycle. Furthermore, C666-1 Nasopharyngeal carcinoma (NPC) cells [33] established from undifferentiated nasopharyngeal carcinoma derived from an NPC xenograft of southern Chinese origin in 1979 are still the only EBV + ve NPC cell lines available for NPC and EBV research. Therefore, progress in this field remains limited. The need to establish new and representative NPC cell lines is crucial for NPC and EBV research.

The major obstacle in the field of virus-mediated studies, including virus-mediated tumorigenesis and virus interaction with the host, is the scarcity of representative cell lines. Moreover, many of the commonly used cell lines are cross-contaminated with HeLa cells [34,35].

Animal models are often used for studies of disease modeling, virus infections, and the development of vaccines and antiviral drugs. In addition, there is considerable animal-to-animal variation, limitations due to species differences, and they are very expensive and time-consuming. This is especially because human viruses often need a series of passages in vivo to adapt to the host environment due to tropism restrictions including variable receptors on the cell surface and may have intracellular restrictions from the cell types or host species [36].

## 3. Current Available Cell Lines

Although accumulated evidence indicated that the same cancer cells may show different characteristics in in vitro cell cultures, in animal models, and in patients [37,38], cell culture models were utilized in human EBV or HPV research. However, these cell lines depended on immortalized epithelial cells harboring episomal or integrated viral genomes. which limited the progress of virus and viral-mediated studies, especially because the efficient infection of primary cell lines is still challenging thus far [38]. Table 1 summarized the most common cell line models for HPV and EBV research.

## 4. Conditional Cell Reprogramming (CCR) at ALI for Modeling Host–Virus Interactions and Virus-Mediated Tumors

CCR is a modern approach for reprograming primary cells, normal or diseased including tumor cells, derived from any organ of the body, which maintain their ability to grow and differentiate with stem-like characteristics, long-term culture, and reversible (or conditional) pattern. CCR involves the co-culture of primary cells with feeder cells, mouse 3T3-J2 fibroblasts inactivated by radiation, and RHO kinase inhibitor, Y-27632.

In ALI, cells are cultured with their basal surfaces submerged in a culture medium and their apical surfaces are exposed to the surrounding air, allowing the cells to differentiate morphologically and functionally mimicking their natural environment [53,54].

The establishment of patient-derived cancer models (PDCMs) as the next generation of cancer models (such as patient-derived xenografts (PDXs) and conditionally reprogrammed cells (CRCs)) is highly needed to increase our knowledge of viral pathogenicity. A promising long-term cell culture system was established to overcome the limitations of conventional long-term cancer cells cultures including development of abnormalities due to genetic manipulation, or genetic and epigenetic changes. With the addition of Y-27632 and feeder layer coculture (conditional reprogramming, CR), CRCs can be propagated efficiently and rapidly from core or needle biopsies, surgical specimens, and other minimally invasive or noninvasive specimens [30,55,56,57]. This technology has wide applications in modeling human viral diseases and drug discovery [24].

### 4.1. CCR Applications in HPV

Since there is a lack of models of natural HPV infection and the complexity of the HPV life cycle, there are no available cell lines from vulvar, anal, or penile lesions and cancers in the field.

Using the CR approach, Wu et al. successfully established a human cell line with naturally infected HPV18 from vulvar intraepithelial neoplasia (VIN) for the first time. Unlike HeLa cells, the VIN cells expressed squamous epithelium-specific markers. When cultured under a 3-D ALI system, the expression of both early and late differentiation markers involucrin and filaggrin, as well as viral late gene L1, were detected in the cornified layer of VIN-ALI 3-D culture derived, suggesting quite a different HPV genomic status from cancer cells [48].

Yuan and colleagues, in 2017, successfully generated and propagated a culture (GUMC-395) of an HPV-16-positive, large cell of extremely rare and aggressive neuroendocrine cervical cancer that was metastasized to the liver [46]. The GUMC-395 cultures hosted HPV-16 and formed colonies in soft agar. It was able to initiate tumors in immunodeficient mice.

CR was used to establish an HPV-6 positive culture from a recurrent respiratory papillomatosis (RRP) patient, named GUMC-403. Alkhilaiwi et al., 2019, conducted high-throughput screening (HTS) to identify candidate drugs to treat this morbid disease [50]. CR HPV-6 cells were used for HTS against >2800 approved drugs for NPC to identify new indications for FDA-approved drugs, as well as against >1900 of mechanism interrogation plate (MIPE) library for novel candidate drugs or investigational drugs at the research and development stage. A total of 13 drugs were identified from the two libraries, with significant lethal effects in CR RRP cells. The results were validated using two-dimensional (2-D) and 3-D cell cultures. The authors suggested three drugs with the potential for future therapies for RRP patients (panobinostat, dinaciclib, and forskolin).

Recently, CR technology was used to establish three radioresistant and two radiosensitive cell lines from cervical cancer patients’ specimens and verified their molecular characteristics and biological changes including growth kinetics, immunofluorescence, clone forming assay xenografting, and immunohistochemistry vitro and in vivo [58]. The results of single-cell RNA (sc-RNA) sequencing analysis showed that 38.1% of cells in radiosensitive CR cell lines aggregated in the G2/M cell cycle phase which is sensitive to radiation compared to 20.83% of cells in radioresistant CR cell lines.

### 4.2. CCR Applications in EBV

Molecular pathogenesis of EBV in epithelial cells is still poorly understood, mainly due to lack of adequate disease models. Two-dimensional in vitro cell culture is inefficient [59]. EBV-infected cells are not abundant in the normal nasopharyngeal epithelium, but they can be detected in nasopharyngeal biopsies [60,61]. This discrepancy may be due to small areas of infection that are difficult to obtain by biopsy sampling methods and/or vigorous immune surveillance. Therefore, a conventional 2-D cell culture is the most common approach to study molecular pathogenesis of EBV in the nasopharynx.

Although EBV latent infection in epithelial cells was heavily investigated using cell culture, not many aspects of the differentiated biology of EBV were captured. Especially because conventional cell culture does not reproduce all the cell types of the nasopharyngeal epithelium [62]. Furthermore, reactivation of conventional 2-D EBV-infected cells is challenging, even when treated with chemical enhancers [63].

Adherent monolayer 2-D cultures can recapitulate EBV latency but not the physiological stimulus for EBV reactivation in epithelial cells, lytic reactivation in response to cellular differentiation [64,65,66]. Three-dimensional culture models are sophisticated and establishing conditions for de novo infection of primary keratinocytes is arguably the most intricate but likely the most authentic model.

Unlike 2-D cell culture, cells in 3-D culture can grow in all directions into spheroids, or into 3-D cell colonies and interact with surrounding in three dimensions. Although 3-D cell culture techniques may have some limitations associated with imaging and incompatibility with flow cytometry and many fluorescence microscopes, they hold more physiologically relevant to in vivo models [67,68].

Yu et al., 2020, successfully infected an established 3-D culture of pseudostratified epithelial cells from one donor with EBV in vitro. The efficient infection was determined by in situ hybridization of BRLF1 and EBER1 using RNAScope [69]. This sensitive method provides a singular detection of EBV transcripts at single cell resolution, which improves the diagnosis of EBV infections. However, it cannot report the active manner (lytic/latent) of EBV infection in biologically reliable form. Ziegler et al. presented a de novo EBV infection model of the nasopharyngeal pseudostratified epithelium from different donors grown in 3-D cell culture from conditionally reprogrammed cells in ALI culture [70]. These models can be used not only to examine the pathogenesis of pre-neoplastic EBV-infected cells, but also to develop anti-EBV therapy or early stage NPC treatment.

It is imperative that we create human respiratory tract-related models including immortalized airway epithelial cell lines, primary airway epithelial cells, lung explants, and cancer cell lines to represent the human respiratory epithelium, which are the primary target cells for this virus. Establishment of these cell lines is not easily achievable, and they deteriorate rapidly in vitro.

Cancer cell lines such as A549 can sustain their growth in vitro, but they no longer mimic the physiological function of human airway epithelium since their phenotype and genetic background were changed [55].

The CR approach overcame this obstacle and was able to expand functional human respiratory epithelial cells rapidly [71]. In EBV research, the absence of NPC representative models significantly restrained the progress. NPC cell lines lose their EBV episomes within 10 to 20 passages of prolonged subculture [52]. Furthermore, the current cell line models are frequently cross contaminated with genetic elements of commonly used HeLa cells [72,73], which limits their application as representative NPC cell lines. Lin et al., 2018, established a transplantation of NPC PDXs specimens in NOD/SCID mice with take rates 4.9% and 17.6% primary and recurrent NPC, respectively. EBV-positive NPC cell line, NPC43, was successfully established from patient NPC tissues by adding Rho-associated coiled-coil containing kinases inhibitor (Y-27632) to culture medium. Withdrawal of Rho inhibitor led to spontaneous lytic reactivation of EBV. The mutational profiles of these CRCs were close to their corresponding patient NPC as shown by whole-exome sequencing. The sequences of EBV genomes were also detected suggesting the potential application of these CRCs [51]. New EBV+ ve NPC cell line, C17, was generated from an earlier established NPC xenograft, using Y-27632 by Yip et al., 2018. The cells showed tumorigenesis in immune-deficient mice (NOD/SCID) with EBV episomes retained associated with lytic reactivation of EBV. This will provide an efficient model to study the effect of lytic and latent infection of EBV and their contributions to NPC initiation [52].

## 5. ALI Cultures for EBV/HPV Biology and Cancer Initiation

### 5.1. ALI Cultures for EBV

EBV infection can cause clonal proliferation of a latently infected cell in the nasopharynx and develop NPC. EBV pathogenesis in nasopharyngeal epithelium is still poorly understood. Moreover, the ability to infect epithelial cells with EBV in vitro is challenging and has variable efficiencies [69]. Little is known about establishment of cell lines from EBV-infected epithelial tumors. It was known for decades that EBV is associated with epithelial malignancies such as gastric carcinoma and subtypes of nasopharyngeal carcinoma [74]. Nevertheless, our knowledge about the life cycle of the virus, lytic reactivation in response to cellular differentiation, and cancer initiation mechanisms is still limited.

Organotypic cultures may represent a better model of EBV-infected epithelial than monolayer cultures because epithelium exists as a stratified tissue in vivo. The use of the ALI method with established CRCs-EBV-infected cell lines may offer an efficient and reproducible approach to study the molecular pathology and permissive replication of EBV in polarized epithelia. ALI approach is more applicable for the study of viral infections, since it provides an open apical approach where the release of viral particles or products, such as: enzymes, cytokines, cellular, or viral genes that contribute to the initiation and progression of NPC, including EBNA1, EBER1/2, LMP1, and LMP2, can be detected and quantified in the lower chambers. The advantage of using CCR cells is that they are easy to obtain from patients to create ALI cultures, which will increase the ability to explore personalized approaches significantly. Moreover, they will minimize experimental variability and cells will grow faster. The variability of ALI models is less than animal models with maintaining the consistency from start to finish. Therefore, this physiological system coupled with CCR significant potential to increase our fundamental knowledge of oncogenic virus infections.

Temple and colleagues, in 2014, demonstrated that organotypic epithelial cell cultures allow the cells to stratify and differentiate as they do in vivo. Organotypic cultures of epithelial cells were efficiently infected with EBV in the suprabasal layers of stratified epithelium [65]. The cells expressed productive-cycle proteins as well as latency-associated proteins but did not induce cellular proliferation. The results suggest that EBV infection of the epithelial cells is a crucial step of the viral production and spread but not the growth or immortalization of the infected epithelial cells. Caves et al., in 2018, used ALI of EBV-infected NPC cells and reported an efficient reactivation of EBV, with production of infectious progeny virus, induction of the lytic cascade, and replication EBV genomes [63]. Ziegler et al. showed that CRCs/NPC at ALI with pseudostratified epithelial cells from nine doners displayed different susceptibility to de novo EBV infection. Although all cultures expressed EBV epithelial cell receptor Ephrin receptor A2, α-tubulin in cilia, and pseudostratified markers MUC5AC, CK7, cultures from two donors expressed EBERs but not the other lytic infection markers. Only cells from one donor yielded lytic infection. Another study aimed to investigate the role of BDLF2 glycoprotein in EBV infection, Walston et al., 2023, generated a Δ*BDLF2* recombinant EBV in which the *BDLF2* gene was replaced with a puromycin resistance gene. Both HEK293 and B cells were infected, primary B cells were also immortalized. The authors infected organotypic cultures with wild type viruses and observed focus of infection represented by numerous clusters of infected cells compared with cells infected with lack of BDLF2 viruses, which displayed fewer infected and isolated cells. This suggested a significant role of BDLF2 in intercellular viral spread [75]. Sciver et al. seeded NOKs-Akata cells over-expressing ΔNp63ɑ onto collagen-treated membranes in ALI culture conditions. These cells had reduced expression of the lytic EBV proteins, R, Z, and BMRF1, concluding that ΔNp63ɑ inhibits lytic reactivation during epithelial cell differentiation [76].

Developed co-infected NOKs cells with EBV and HPV at ALI and indicated that HPV promoted lytic reactivation of EBV in upper layers of stratified epithelium and increased maintenance of the EBV genome [77].

### 5.2. ALI Cultures for HPV

Several studies used ALI epithelial cultures to study HPV, since viral replication is restricted to the differentiation of the host epithelial cells. Some HPVs are associated with intraepithelial neoplasia (vaginal, penile, vulvar, or cervical) and epidermodysplasia verruciformis [78]. A generation of organotypic raft cultures formed a squamous epithelium that mimicked the original tissue, which achieved some progress in HPV research [79,80,81].

Remarkably, this system was able, for the first time, to produce the infectious HPV virions [82]. Epithelial raft cultures were further used to study HPV biology by other researchers [83,84,85]. Moreover, autonomous HPV-18 genomes were generated efficiently in organotypic raft culture of human keratinocytes [64,86].

In 2001, Delvenne and colleagues established an organotypic (raft) model to study the immunotherapeutic approaches for mucosal surface carcinomas at ALI on a dermal equivalent support. The approach displayed a dysplastic morphology such as neoplastic lesions seen in vivo. Moreover, the system showed ability to be manipulated by integrating dendritic cells or activated lymphocytes into epithelial sheets that form in vitro, as well as by altering the epithelial stratification with cytokines [87].

Bienkowska-Haba et al., 2018, transferred the infected extra cellular matrix (ECM) of HaCaT cells to the primary keratinocytes at ALI system. They observed an efficient uptake and complete HPV16 life cycle following infectious delivery. Replication foci were detected in the HPV16-infected cells; moreover, they expressed L1 proteins and late E1/E4, suggesting the completion of the viral life cycle. Viruses with mutation in E1, E6, and E7 translation termination linker were analyzed for genome maintenance and establishment of infection, which showed that only E1 was essential to establish infection, and E6 was required for episomal genome maintenance [38]. Using this model, they were able to identify transcriptional changes attributable to HPV16 infection by genome-wide transcriptome analysis and illustrated that most perturbed genes belong to S-phase genes, which are regulated by pocket proteins [88]. Furthermore, they demonstrated that most HPV genomes failed to tether to the mitotic chromosomes during mitosis, degraded in G1 phase, which causes genome copy number to reset. Instead, HPV genome was amplified in each S phase [89]. Deng et al., 2019, presented an optimized organotypic culture method to compare growth and differentiation of cells cultured from ectocervix, endocervix, and transformation zone of cervical epithelial cells using collagen rafts with human cervical stromal cells [90]. Bedard and colleagues, in 2021, established HPV+ patient-derived 3-D organotypic epithelial rafts model of juvenile-onset recurrent respiratory papillomatosis (JoRRP). Cells showed high proliferation compared to controls. This model mimicked its patient’s biopsy-derived phenotypes where basal cells proliferated and replicated the suprabasal cells showing low growth at subconfluency, with a switch to high growth after reaching confluency, suggesting a crucial function of cell–cell contact and/or differentiation [91].

Zhou et al., 2022, PCR amplified canine papillomavirus type 1 genome in canine corneal cells and proved its circular episome form by rolling circle amplification assay. They also established the first canine corneal cell by CCR. The cells grew rapidly in vitro maintaining long-term expansion with epithelial cell morphology and, more importantly, maintaining CPV1 genome during long propagation [92]. Coursey and McBride, in 2021, described a protocol to transfect HPV viral DNA into keratinocytes cells using electroporation. They used colony-forming assay to determine the efficiency of HPV extrachromosomal genomes establishment in cells and Southern blotting to measure the copy number status [93].

Li et al. established sixteen head and neck squamous cell cancer (HNSCC) cultures at a tertiary care center in the Bronx using CCR approach. Cell lines were derived from HPV-positive tumors arising in anatomical sites typical of HNSCC. These tumors were established from a series of patients from diverse ethnic populations. Nine cultures of this robust panel were derived from the oral cavity; four and three cultures were derived from the oropharynx and laryngeal carcinomas, respectively. HPV was maintained in three of four oropharyngeal tumors after examination using clinical p16 staining and RT-PCR of HPV16/18. Moreover, the authors reported that CR cultures were able to establish 3-D spheroid, orthotopic tongue models, and murine flank efficiently from patients regardless of their characteristics, molecular background, or disease site [49].

For the first time, CR was used to establish efficiently the first human cell line infected naturally with HPV18 from VIN by Wu et al., 2021. When cultured under 3-D at ALI system, the expression of both early and late differentiation markers involucrin and filaggrin, as well as viral late gene L1, were detected in the cornified layer of VIN-ALI, suggesting different HPV genomic status from cancer cells [48]. Therefore, CCR/ALI will provide a powerful and unique biological or functional cell approach for viral phenotypical and mechanism studies including viral biology, viral–host interaction, the modulators of the host’s innate immune response, as well as viral tumorigenesis. This is especially because CRCs are easily applicable for genetic manipulation [94].

Although a combination of ALI and CRC needs further optimization, it will provide a robust human-relevant physiological system for oncogenic viruses.

## 6. Future Overview

### 6.1. Study the Biology of Oncogenic Viruses

**Figure 1:**
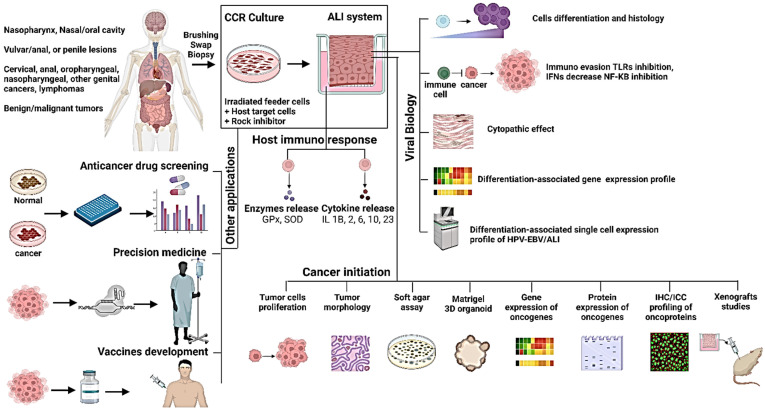
Potential applications of CR/ALI technologies in virus-mediated cancers illustrating suggested key tests to be applied to study viral biology and cancer initiation.

ALI system is a robust approach for the study of viral infections because of an open apical culture system where the expression of cellular or viral genes and the release of cytokines and enzymes occur. Moreover, viral particles or products can be detected or measured from both upper and lower compartments. CR can be used for the generation of HPV-positive benign or malignant lesions in order to investigate the biology of HPVs through native viral infections or HPV DNA transfections in CR host epithelial cells. Papillomavirus E6 and E7 proteins were suggested to enable viral genome replication [95], Brd4 can enhance replication by concentrating viral processes in specific regions of the host nucleus [96], and the contribution of Sp100 in regulating the viral infection [97] can be further investigated. Moreover, the viral life cycle as well as differentiation-associated cellular and viral gene expression could be also revealed using sc-RNA sequencing of HPV/ALI culture (Figure 1).

### 6.2. Study the Potential Role of Oncogenic Viruses in Tumorigenesis

Further studies may be performed to obtain more knowledge about the potential pathways of oncogenic viruses in cell immortalization including cytoskeleton and p16/Rb pathway or induction of telomerase. Additionally, studies looking at the expression of EBV genes including EBNA1, EBER1/2, LMP1, and LMP2 and their contribution to the initiation and progression of NPC are possible. Additionally, the potential effect of papillomavirus E6 and E7 proteins as well as Brd4 to enhance tumorigenesis can be investigated using CCR/ALI models. EBV/ALI culture may provide an adequate system to study the EBV life cycle and differentiation-associated viral and cellular gene expression using sc-RNA sequencing (Figure 1).

### 6.3. Anticancer Drug Screening

Recently, CR cells were employed in clinical and translational research for drug discovery. CR cells were obtained from different regions of each tumor of four patients with renal cell carcinoma (RCC), and HTS was applied using 306 clinical and emerging cancer drugs. The results demonstrated sensitivity in the CR RCC cells to conventional RCC drugs [50]. CR was also used to grow primary cells from a rare salivary gland cancer by Chen et al. (2017). Regorafenib was identified and suggested as a potential therapeutic drug [98]. Recently, Alamri et al. (2018) showed that allosteric the AKT inhibitor MK2206 can inhibit the growth of mucoepidermoid carcinoma (MEC) cells in 2-D and 3-D CR cultures [99]. Therefore, CR has great potential for drug discovery systems to validate drug sensibility and IC50s. It can also be utilized to discover novel drug-resistant mechanisms targets while accelerating target identification and validation, given that the gene expression patterns found in 3-D models can better mimic physiological conditions (Figure 1).

### 6.4. Anti-Viral Screening

In addition to studying virus infections and interactions with host cells, CRC-coupled with ALI technologies may serve as ex vivo physiological models for antivirus drug screening and evaluation of drug efficiency and toxicity. This will facilitate studies of virus entry, innate immune responses, viral replications, and drug discovery. It was used to study the antiviral drugs against Zika virus and found that Arbidol (ARB, umifenovir) inhibited six different isolates of Zika virus including Asian and African lineage viruses [100]. Therefore, CR may provide a better understanding of virus–host interaction and serve as a physiological model for antiviral discovery (Figure 1).

### 6.5. In Precision Oncology

Cancer cell lines usually lose their cellular complexity and heterogeneity of human cancers. The current available cancer cell lines may not reflect the genetic background or racial and ethnic groups of the patients where they are derived, or the spectrum of cancer cell types of their origin. This may explain why precision medicine based on genomic information benefits less than 20% of patients with solid tumors [101]. Cancer cells have a distinct physiology from normal cells which influences sending or receiving the signals to or from their adjacent cells. Recently, CR culture showed an ability to maintain and imitate the ordinary biology of their primary tissue such as tracheal epithelium, ectocervical epithelium, or breast tumors [30,102]. Additionally, intra-tumoral heterogeneity was maintained in CR cells, suggesting oligoclonality of these cultures [103]. CR from individual patients will provide information on predicted responses to medications and allow more precise evidence which contributes to a better understanding of cancer biology, the discovery of novel biomarkers, and novel drug targets (Figure 1).

### 6.6. Cancer Vaccine Development

Many markers can be investigated to obtain more knowledge about the host immune response including GPx, SOD, and ILs, which can be released to lower compartment, as well as markers related to viral immune evasion, such as TLR, IFNs, and NF-KB.

Animal models are often used for disease modeling, virus research, development of antiviral/anticancer drugs, and vaccines. In order to adapt to the host environment, human viruses often need a series of passages in vivo because of variable receptors on the cell surface and may have intracellular restrictions from the cell types or host species. CR can be used for the generation of HPV or EBV benign or malignant human lesions, anti-HPV/EBV discovery, and biology of both high- and low-risk HPVs and EBV through native viral infections or HPV/EBV DNA transfections in CR host epithelial cells. CR as a long-term cell culture system may hold great potential applications in modeling human viral diseases and vaccine discovery.

Since CR cells maintain their lineage functions, it provides biologically and physiologically relevant models, which are suitable for the study of cancer antigens, viral entry and replication, innate immune responses of host cells against virus-mediated cancers, and the discovery of antiviral or anticancer vaccines.

## 7. Conclusions

There is an urgent need for human physiological cell models to facilitate the study of virus-associated human carcinomas, human viral disease, and host–virus interactions. Here, we summarized the applicability of CRC-coupled ALI technology to serve as ex vivo physiological models and to overcome the limitations of the current cell lines and animal models.

As we summarized in Figure 1, CCR/ALI technology is a robust and rapid long-term culture system, where the cells can be established from a wide variety of specimens. Therefore, it holds great promise in modeling human virus-associated carcinomas to study viral life cycle, cancer initiation, viral diseases, anti-viral drug screening, and cancer vaccine development.

## Figures and Tables

**Table 1:** List of the most common cell line models for HPV and EBV research.

Cells	Source	Virus Type	Integrated/ Episomal	Reference
HeLa	Cervical carcinoma	HPV18	integrated	[39]
SNU-1000	Cervical squamous cell carcinoma isolated from a 43-year-old Korean patient	HPV16	episomal	[40]
SNU-1245	Cervical squamous cell carcinoma	HPV18	episomal	[40]
W12-20850	low-grade squamous intraepithelial lesion	HPV16	episomal	[41]
CIN612	Cervical intraepithelial neoplasia	HPV31b	episomal	[42]
SiHa	Squamous cell carcinoma of the cervix uteri	HPV16	integrated	[43,44]
CaSki	Cervical squamous cell carcinoma	HPV16	integrated	[43,45]
SCC-090	Tongue squamous cell carcinoma	HPV16	integrated	[43]
SNU-1245	Squamous cell carcinoma of the cervix uteri	HPV18	integrated	[40]
GUMC-395	Neuroendocrine cervical cancer	HPV16 E6 and E7	integrated	[46]
Chlamydia and HPV co-infection cells	Cervical epithelium	HPV16 E6 and E7	integrated	[47]
Vulvar intraepithelial neoplasia (VIN) cells	Vulvar intraepithelial neoplasia	HPV18	episomal	[48]
HNSCC	Head and neck squamous cell cancer	HPV16/18	episomal	[49]
GUMC-403	Lung tissue of RRP patient	HPV-6	episomal	[50]
NPC43	Nasopharyngeal carcinoma	EBV	episomal	[51]
C666-1 NPC	Nasopharyngeal carcinoma	EBV	episomal	[33]
C17 EBV + ve NPC	Nasopharyngeal carcinoma	EBV	episomal	[52]

## Data Availability

Not applicable.
